# On the biospheric effects of geomagnetic reversals

**DOI:** 10.1093/nsr/nwad070

**Published:** 2023-03-13

**Authors:** Yongxin Pan, Jinhua Li

**Affiliations:** Key Laboratory of Earth and Planetary Physics, Institute of Geology and Geophysics, Chinese Academy of Sciences, China; Key Laboratory of Earth and Planetary Physics, Institute of Geology and Geophysics, Chinese Academy of Sciences, China

## Abstract

This perspective argues an evolutionary effect of geomagnetic field reversals on life and highlights the urgency of multidisciplinary studies on the linkage between Earth's magnetic field and biosphere.

Unlike its neighbouring planets Mars and Venus, Earth has a global magnetic field. The geomagnetic field effectively protects life from the solar wind and cosmic radiation, prevents atmospheric erosion and water loss, and thus Earth makes for a habitable planet [1]. The geomagnetic field also provides useful clues to orientation and navigation for a diverse group of organisms, from bacteria to vertebrates. Ground station and satellite measurements indicate that the strength of the present-day magnetic field is decreasing and the South Atlantic Anomaly, a huge area spanning South Africa to Patagonia of low field strength, is continuously growing in size. These raise concerns and discussion both among specialists and the public on whether a geomagnetic field reversal—a flipping with the pole reversing signs—may be imminent; if this happened, life on Earth, including for us humans, may face high irradiation and other environmental risks.

Paleomagnetic studies have indicated that the geomagnetic field has reversed its polarity at least several hundred times during the Phanerozoic eon. The strength of the dipole field can decrease by as much as 90% at Earth's surface during a reversal or an excursion (also called a failed/aborted reversal) (Fig. 1a)and b). Recently, Channell and Vigliotti found that the timing of geomagnetic field strength minima across Quaternary geomagnetic excursions appears to correspond to events in mammalian evolution [2]. Ueno and co-workers proposed that an increase in galactic cosmic rays during the Matumaya–Brunhes transition (the last geomagnetic polarity reversal at ∼780 ka ago) produced an ‘umbrella effect’ of low cloud cover that led to high atmospheric pressure in Siberia, possibly causing the East Asian winter monsoon to become stronger [3]. In deeper time, a good opportunity to uncover the impacts of geomagnetic field variation on the biosphere is the intervals of high-frequency geomagnetic reversals (Fig. 1b). Meert and co-workers further suggested that the high-frequency geomagnetic reversals during the Ediacaran–Cambrian transition might be responsible for a major replacement of biota that led to the appearance of Phanerozoic higher-level taxa and ecological communities in the marine realm [4]. Recently, Tarduno's group contributed single-crystal paleointensity data and found the Ediacaran time-averaged field strength to be extraordinarily low [5], followed by a significant increase in strength in Cambrian. Nevertheless, Lingam argued that the dynamic and heterogeneous Ediacarian geochemical environment, rather than the nucleation of the inner core or an increase in the magnetic field strength, appears more plausible in driving the Cambrian radiation [6].

**Figure 1.**
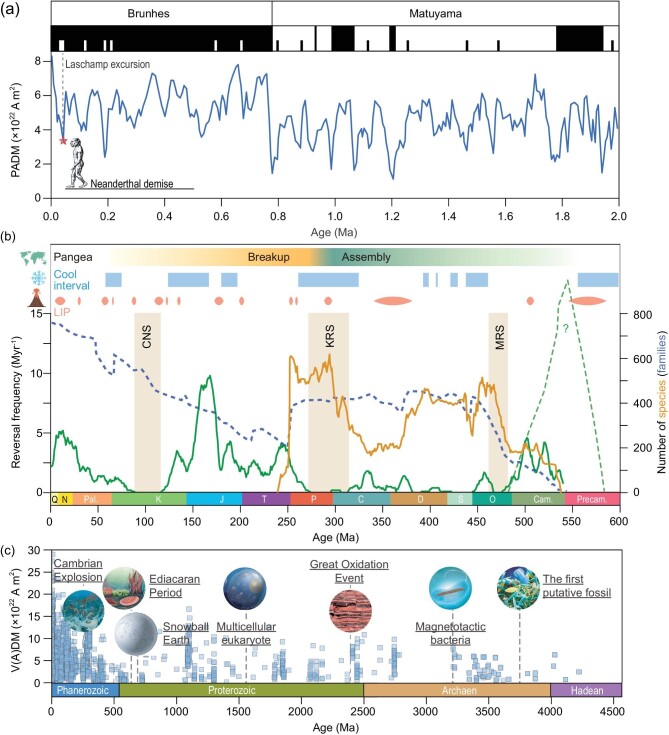
(a–c) Polarity, reversal frequency and strength of the geomagnetic field correlate with major geological and biological events (see the Supplementary materials online for detailed description).

Indeed, as one of the important prerequisites, the geomagnetic field is a constructive factor enabling life on Earth: adding protection against atmospheric escape and diminishing doses of ultraviolet radiation (UVR), galactic cosmic rays and other energetic particles reaching the surface. Moreover, as a factor in environmental change on an enormous scale, geomagnetic reversals could impact many elements of the biosphere. Specifically, in this scenario, geomagnetic field reversals can have a positive evolutionary effect by stimulating the emergence of new function and new organisms via associated environmental changes. Paleomagnetic records indicated that the geomagnetic field might exist since the late Hadean, prior to the development of the first microbial life (Fig. 1c) [1]. Although the radiative effects of energetic charged particles of solar or cosmic rays may not directly negatively impact the biosphere because of shielding by the atmosphere, their increasing entry into the atmosphere during geomagnetic field transitions can lead to an increase in ozone loss and an increasing penetration of solar UVR to Earth's surface [7]. On the one hand, solar UVR can produce a multitude of harmful effects on cells including alteration in the structures of DNA and other biologically relevant molecules, chronic depression of key physiological processes and acute physiological stress. These can act as a kill mechanism for terrestrial life. On the other hand, UVR stress might have been one of the major ambient selective pressures that led to life developing a wide range of evolutionary adaptations to defend against harmful UVR. These include behavioural mechanisms (e.g. migrations, burrowing, nocturnal activity), screening or absorption (i.e. pigments such as mycosporine-like amino acids, carotenoids or melanin) and cellular mechanisms (i.e. DNA repair, antioxidants and biomineralization). UVR stress would bring about increases in the mutation rates within populations as a barometer of speciation, which is the foundation of biodiversity and evolution. Thus, aperiodic yet frequent geomagnetic field reversals, rather than merely killing mechanisms, could be an evolutionary force speeding up mutation rates, speciation and biological innovation through environmental UVR stress.

Life on Earth has been the result of a continuous, yet variable, selection since its onset. Evolution is composed of many events of mass extinctions, recoveries and radiations that were often interactively coupled with significant environmental perturbations (Fig. 1a–c). Many organisms have developed evolutionary adaptations to co-evolve with Earth's environmental disturbance that includes changes in the geomagnetic field (e.g. geomagnetic reversals, superchrons) and associated UVR flux. Thus, the geomagnetic field is a natural component of environmental forcing that is important for organisms. Interestingly, from the very beginning of their existence, organisms functioned and evolved in the presence of the geomagnetic field. By using the geomagnetic field as a source of spatial information, many organisms, from magnetotactic bacteria (MTB) to plants and animals, could better adapt to environmental and climate changes. Examples include long-distance animal migration/navigation and up-and-down MTB magnetotaxis shuttle [8,9]. We recently found that mice experiencing long-term exposure to hypomagnetic fields exhibit significant impairments of adult hippocampal neurogenesis and hippocampus-dependent learning, implying that the geomagnetic field is essential for mammals [10]. In light of ever-accumulating evidence, we conclude that geomagnetic field effects on the biosphere have always existed, and that these directly and indirectly have influenced biotic evolution. These have acted together with other global environmental processes such as sea-level fluctuation, paleoclimate change, plate tectonics, volcanic eruptions, oxygenation, asteroid impact and other processes. But the effects of the geomagnetic field have been non-negligible throughout the history of life on the planet.

Therefore, it is necessary to gauge the biospheric effects of geomagnetic field variations in the space–time dimension. So far, our understanding of the fundamental mechanisms between geomagnetic fields and biological effects is incomplete due to the complexity of the linkages and shortage of data. Multidisciplinary studies on the geomagnetic field–UVR–life interplay are crucial for a better understanding of cause–effect relationships. A combination of behavioural, physiological and molecular studies could provide insights into the fundamental mechanisms. Simulation and experimental studies on the geomagnetic field–atmosphere and climate interplay are also needed to assess whether changes in the geomagnetic field could trigger the changes in cloud cover and precipitations—topics that are currently intensively debated. Future studies recovering records of the paleomagnetic field and ancient life in rocks (integrated paleoenvironmental, paleomagnetic and paleontological studies, including big data analyses) and, in particular, studies of specific polarity transitions and/or high-frequency geomagnetic reversal intervals are needed to decipher deep-time truth. Such investigations could provide unprecedented insights into understanding the linkage between the geomagnetic field and the biosphere, the co-evolution of life with environments and, ultimately, planetary habitability.

## Supplementary Material

nwad070_Supplemental_File
