# Supplementary material for: On the biospheric effects of geomagnetic reversals
Source: Natl Sci Rev. 2023 Mar 13;10(6):nwad070. doi: 10.1093/nsr/nwad070 (PMC10171621; doi:10.1093/nsr/nwad070)
Supplement: nwad070_Supplemental_File [file nwad070_supplemental_file.docx]

Supplementary Materials for

**On the biospheric effects of geomagnetic reversals**

Yongxin Pan*, Jinhua Li

Key Laboratory of Earth and Planetary Physics, Institute of Geology and Geophysics, Chinese Academy of Sciences, Beijing 100029

*Corresponding author. Email: yxpan@mail.iggcas.ac.cn

**This file includes the detailed description of Figure 1 in main text.**

Figure 1: (a) Geomagnetic polarities, paleointensity variations, and validated geomagnetic excursions for 0-2 Ma. Black (white) represents normal (reverse) polarity. The blue line represents the paleointensity variations based on the paleomagnetic axial dipole moment (PADM) model [1]. (b) Correlation number of species (family), the reversal frequency of geomagnetic field and other major geological events. Solid green line, the reversal frequency, which was computed with a sliding window of 5 Ma based on the geomagnetic polarity time scale [2]; Dashed green line, the reversal frequency modified from [3]. Orange line and dashed blue lines, the number of species and families from [4] and [5], respectively. The large igneous provinces (LIP) data are from [6]. The cool interval and Pangea supercontinent configuration data are from [7] and [8], respectively. CNS, Cretaceous normal superchron. KRS, Kiaman reversed superchron. MRS, Moyero (Ordovician) reversed superchron. (c) Relationship between geomagnetic field strength and biological radiation events. Solid blue squares show the available paleointensity data from [9, 10]. The color spheres, from right to left, represent the first putative fossil, the origin of magnetotactic bacteria, Great Oxidation Event, multicellular eukaryote, Snowball Earth, Ediacaran Period, and Cambrian Explosion.

1. Ziegler LB, Constable CG, and Johnson CL *et al*. PADM2M: a penalized maximum likelihood model of the 0-2 Ma palaeomagnetic axial dipole moment. *Geophys J Int* 2011; **184**: 1069-1089.
2. Gradstein FM, Ogg JG, and Schmitz MD *et al*. *Geologic Time Scale* 2020. Elsevier.
3. Meert JG, Levashova NM, and Bazhenov ML et al. Rapid changes of magnetic field polarity in the late Ediacaran: linking the Cambrian evolutionary radiation and increased UV-B radiation. *Gondwana Res* 2016; **34**: 149-57.
4. Fan J, Shen S, and Erwin DH, *et al*. A high-resolution summary of Cambrian to Early Triassic marine invertebrate biodiversity. *Science* 2020; **367**: 272-277.
5. Raup DM and Sepkoski JJ. Mass extinctions in the marine fossil record. *Science* 1982; **215**: 1501-1503.
6. Bond DP, Wignall PB, and Keller G *et al*. Large igneous provinces and mass extinctions: An update. *Volcanism, impacts, and mass extinctions: Causes and effects* 2014; **505**: 29-55.
7. Scotese CR, Song H, and Mills BJ *et al*. Phanerozoic paleotemperatures: The earth’s changing climate during the last 540 million years. *Earth Sci Rev* 2021. **215**: 103503.
8. Scotese CR. An Atlas of Phanerozoic Paleogeographic Maps: The Seas Come In and the Seas Go Out. *Annu Rev Earth Planet Sci* 2021; **49**: 679-728.
9. Bono RK, Paterson GA, and Biggin AJ. MCADAM: A continuous paleomagnetic dipole moment model for at least 3.7 billion years. *Geophys Res Lett* 2022; **49**: e2022GL100898.
10. Tarduno JA, Cottrell RD, Bono RK et al. Paleomagnetism indicates that primary magnetite in zircon records a strong Hadean geodynamo. *Proc Natl Acad Sci* 2020; **117**: 2309-18.
